# Integrative analysis of summary data from GWAS and eQTL studies implicates genes differentially expressed in Alzheimer’s disease

**DOI:** 10.1186/s12864-022-08584-8

**Published:** 2022-06-02

**Authors:** Brian Lee, Xiaohui Yao, Li Shen, for the Alzheimer’s Disease Neuroimaging Initiative

**Affiliations:** Department of Biostatistics, Epidemiology and Informatics, Perelman School of Medicine at the University of Pennsylvania, Philadelphia, USA

**Keywords:** GWAS, eQTL, Transcriptomics, Alzheimer’s Disease

## Abstract

**Background:**

Although genome-wide association studies (GWAS) have successfully located various genetic variants susceptible to Alzheimer’s Disease (AD), it is still unclear how specific variants interact with genes and tissues to elucidate pathologies associated with AD. Summary-data-based Mendelian Randomization (SMR) addresses this problem through an instrumental variable approach that integrates data from independent GWAS and expression quantitative trait locus (eQTL) studies in order to infer a causal effect of gene expression on a trait.

**Results:**

Our study employed the SMR approach to integrate a set of meta-analytic cis-eQTL information from the Genotype-Tissue Expression (GTEx), CommonMind Consortium (CMC), and Religious Orders Study and Rush Memory and Aging Project (ROS/MAP) consortiums with three sets of meta-analysis AD GWAS results.

**Conclusions:**

Our analysis identified twelve total gene probes (associated with twelve distinct genes) with a significant association with AD. Four of these genes survived a test of pleiotropy from linkage (the HEIDI test).Three of these genes – RP11-385F7.1, PRSS36, and AC012146.7 – have not yet been reported differentially expressed in the brain in the context of AD, and thus are the novel findings warranting further investigation.

## Background

Alzheimer’s disease (AD) is a complex neurodegenerative disease commonly characterized by memory impairments, cognitive problems, and the presence of both tau and A *β* plaques [1]. As the leading cause of dementia, AD is influenced by environmental and genetic factors [2]. There is no current cure for AD, necessitating larger-scale approaches.

Since genetic factors play an important role in AD, genome-wide association studies (GWAS) have been employed to find specific loci and genes that may be instrumental in both AD treatments and prognosis. So far, GWAS has successfully identified numerous loci susceptible for AD [3]. However, translating these findings has proven extremely difficult. GWAS provides insights into potential genetic risk loci likely to harbour causal variants. Despite having multiple analytical techniques including fine-mapping, advanced annotation tools, and colocalization, difficulties remain in inferring which variants are truly causal in AD. Understanding the mechanisms by which these variants influence disease phenotypes including AD provides additional challenges [4]. These challenges arise from factors such as complex linkage disequilibrium and potential effects on distant genes. Additionally, the dynamic, context-specific effect of variants are likely to vary depending on the time, cell type, and the context being studied.

In addition to direct genetic analyses, studying gene expression of AD-relevant genes may provide more information about the mechanism of AD. Unfortunately, however, this is extremely difficult as there is a lack of in-vivo Alzheimer’s studies involving human brain tissue. As such, we resort to data from landmark projects such as the Genotype-Tissue Expression (GTEx) project [5] – an ongoing effort to build a comprehensive public resource to study tissue-specific gene expression and regulation. Researchers can now access increasingly large amounts of valuable information that connect significant variants with the expression of specific genes in various tissues. The findings that make up these datasets are often referred to as expression quantitative trait loci (eQTL). Various projects including the GTEx project and ROS/MAP [6, 7], which refers collectively to both *the Religious Orders Study* and *the Rush Memory and Aging Project*, find and store significant eQTL’s for several tissues throughout the human body, including the brain. However, almost none of this information incorporates knowledge currently known about pathologies or diseases – including AD – in highlighting specific genes or variants. Currently, many GWAS hits for diseases including AD reside in intronic or intergenic regions and as such may not make attractive druggable targets. Outside of rare missense or nonsense coding variants, moving from GWAS findings into druggable targets has not proven extremely successful. As such, integrating eQTL studies with previous GWAS hits may prove to be more successful. With the advent of Summary-Data-Based Mendelian Randomization (SMR), it is possible to employ an instrumental variable approach in integrating independent GWAS and eQTL studies [8]. Doing so is especially powerful in that it allows for researchers to find specific genes with a strong functional component in the context of a specific disease – e.g., Alzheimer’s. Through this analytical technique, we aim to identify novel genes that are differentially expressed in AD, which may help reveal the biological pathway from genetic determinants to transcriptomic features to phenotypic outcomes and help disease modeling and therapeutic target discovery.

## Results

Using the above specified ADNI genotyping data, three sets of meta-analytic GWAS summary statistics, and one set of meta-analysis cis-eQTL information, three SMR analyses were performed. Given that each SMR analysis reports the significance of each proposed gene-phenotype association in terms of a *P*-value, a standard Bonferroni correction was used to determine significance given the occurrence of multiple trials. For each analysis performed, given the varying number of relevant SNP’s and gene expression probes that passed the program’s strict eligibility thresholds, the Bonferroni correction was determined by the number of gene probes tested per analysis. As such, this threshold fluctuated slightly among the three analyses, and is as follows: for the SMR based on Lambert et al., 2013 [9], the threshold is 6.90×10^−6^; for the SMR based on Jansen et al., 2019 [10], the threshold is 6.89×10^−6^; for the SMR based on Kunkle et al., 2019 [11], the threshold is 6.85×10^−6^.

**Fig. 1:**
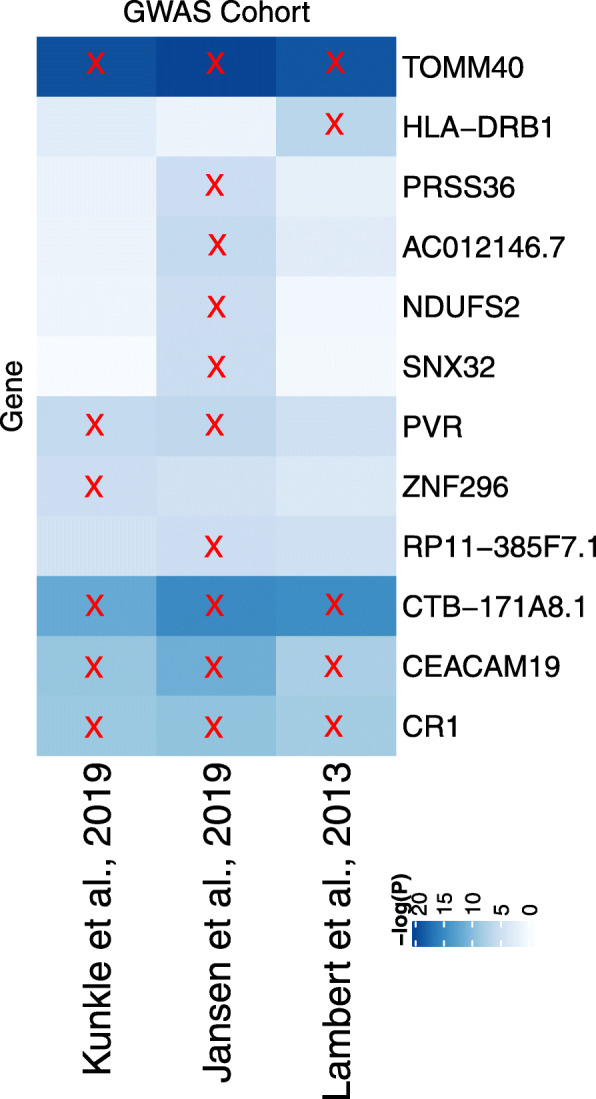
This heatmap shows the *p*-values of our SMR analyses. Along the x-axis are the three GWAS studies implemented in our GWAS; along the y-axis are the genes with associations to our phenotype (AD diagnosis) that have survived the corresponding Bonferroni significance thresholds. The heatmap is employing a negative logarithmic scale

Figure 1 shows a heatmap visualizing our statistically significant findings. Our analysis highlighted 12 gene probes linked to 12 distinct genes between the three summary GWAS studies using the single meta-eQTL. Some findings, such as TOMM40 and CR1, have been explicitly studied as top AD genes. For reference, we also wish to examine the significant GWAS and eQTL relationships that lead to these significant SMR results. We start by comparing the GWAS *p*-values and eQTL *p*-values for each of our twelve significant genes and the SNP with the highest eQTL and GWAS *p*-values that is less than 1 Mb away from the gene (Table 1).

**Table 1:** This table shows the relevant cis-eQTL and summary GWAS *p*-values factored in to our SMR analysis. The index/ leftmost column includes both the gene analyzed along with the SNP with the strongest associations with both gene expression and our AD phenotype; these are the gene and SNP directly analyzed via SMR via the instrumental variables estimation. The first data column (denoted cis-eQTL^a^) contains the **cis-eQTL*p*-values** used from [29]; the final three data columns (denoted GWAS^b^, GWAS^c^, and GWAS^d^) contain the **summary GWAS*p*-values** used (from [11], [10], and [9], respectively); note these are *different*

Gene	SNP	cis-eQTL^a^	GWAS^b^	GWAS^c^	GWAS^d^
*PVR*	*rs11540084*	2.57E-30	5.12E-8	1.87E-8	1.90E-6
*TOMM*40	*rs7259620*	4.05E-22	4.99E-148	5.78E-216	3.25E-125
*NDUFS*2	*rs4379692*	4.12E-19	3.02E-2	7.84E-8	8.07E-2
*ZNF*296	*rs8100183*	4.81E-11	4.52E-10	2.21E-8	8.25E-6
*SNX*32	*rs17854357*	<1.00E-300	3.50E-1	3.12E-6	1.33E-1
*PRSS*36	*rs1549299*	3.36E-18	1.14E-2	6.87E-8	3.21E-3
*CEACAM*19	*rs714948*	7.00E-20	1.35E-16	1.14E-25	6.26E-13
*HLA*−*DRB*1	*rs9271069*	1.79E-95	1.10E-3	2.26E-2	7.53E-8
*CR*1	*rs679515*	2.10E-18	1.55E-16	6.83E-19	4.10E-15
*AC*012146.7	*rs73976310*	6.19E-31	2.14E-2	6.50E-8	5.92E-4
*CTB*171*A*8.1	*rs55710026*	<1.00E-300	9.32E-13	5.59E-16	8.00E-16
*RP*11−385*F*7.1	*rs9473119*	2.67E-13	1.87E-7	1.02E-8	4.59E-8

Of note, we are more interested in identifying pleiotropic associations, where the same underlying causal variant affects the gene expression and the trait. In contrast, we are less interested in the LD-based associations, which could also be detected by SMR. In these associations, the relevant cis-eQTL is in LD with one causal variant affecting gene expression and the other affecting the trait. Thus, to confirm the significance of our results and test for a pleiotropic association versus a LD-based association, we performed a HEIDI test using a *p*-value threshold of 0.05 as used in [8]. Out of the twelve original genes highlighted, we detected heterogeneity for eight genes with *P*_HEIDI_<0.05. The four remaining genes passed the HEIDI test, leading us to not reject the null hypothesis that there is a single causal variant affecting both gene expression and the AD diagnosis outcome phenotype. Hence, these four remaining genes – NDUFS2, RP11-385F7.1, PRSS36, and AC012146.7 – are the most functionally relevant genes underlying the GWAS hits and may be prioritized in future functional studies.

**Fig. 2:**
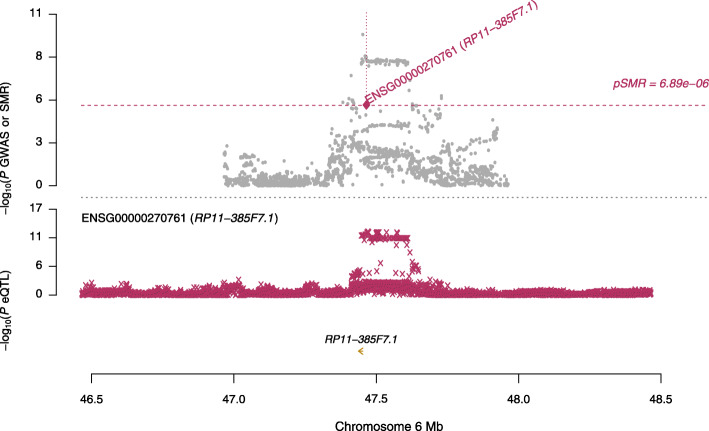
A locus plot showing the significant gene RP11-385F7.1, its location within chromosome 6, and the negative log of the significant *p*-values instrumental in deeming this locus significant in the SMR analysis using Qi et al., 2018 meta-analysis eQTL data and Jansen et al., 2019 GWAS data. The SMR *p*-value noted in this visualization for the gene RP11-385F7.1 is 6.61×10^−6^. Y-axis represents the negative log of the *p*-values; x-axis represents BP location

**Fig. 3:**
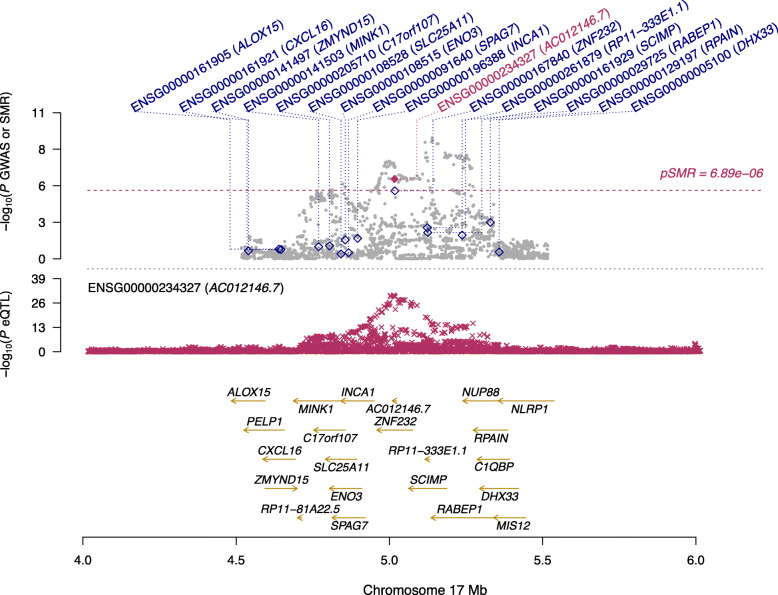
A locus plot showing the significant gene AC012146.7, its location within chromosome 17, and the negative log of the significant *p*-values instrumental in deeming this locus significant in the SMR analysis using Qi et al., 2018 meta-analysis eQTL data and Jansen et al., 2019 GWAS data. The SMR *p*-value noted in this visualization for the gene AC012146.7 is 9.77×10^−7^. Y-axis represents the negative log of the *p*-values; x-axis represents BP location

**Fig. 4:**
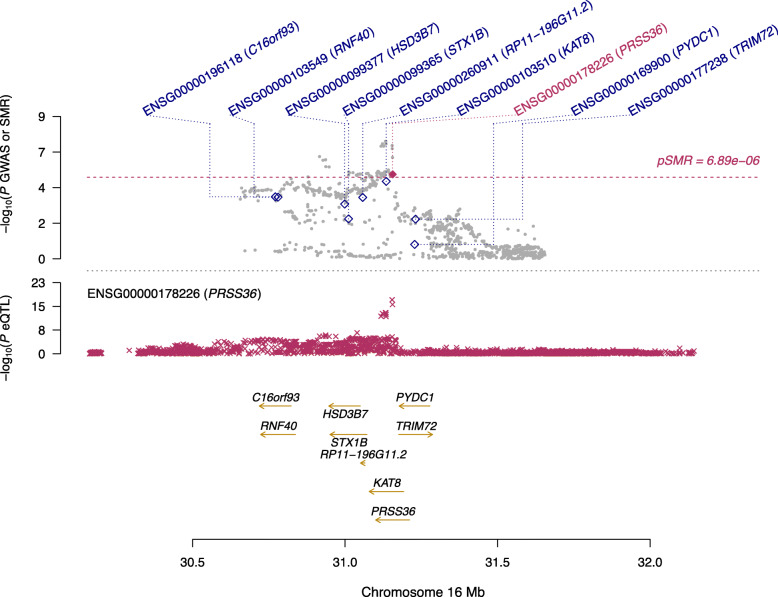
A locus plot showing the significant gene PRSS36, its location within chromosome 16, and the negative log of the significant *p*-values instrumental in deeming this locus significant in the SMR analysis using Qi et al., 2018 meta-analysis eQTL data and Jansen et al., 2019 GWAS data. The SMR *p*-value noted in this visualization for the gene PRSS36 is 4.55×10^−6^. Y-axis represents the negative log of the *p*-values; x-axis represents BP location

Additionally, we searched multiple sources to determine the roles these four genes may play in leading to AD or other diseases. As such, we initially attempted to discover if these genes have been previously declared to be differentially expressed in the brain in relation to AD in the studies [12–14]. The gene NDUFS2 was reported as differentially expressed in [14]. The other three genes have never been reported differentially expressed in Alzheimer’s: RP11-385F7.1, PRSS36, and AC012146.7. These novel findings warrants further replication studies in independent cohorts. To visualize the results of our SMR analysis, we created locus plots for the above three novel findings: RP11-385F7.1 (Fig. 2), AC012146.7 (Fig. 3), and PRSS36 (Fig. 4). These three figures show that the SMR and eQTL *P*-values instrumental in highlighting the significance of these genes in AD in particular.

**Fig. 5:**
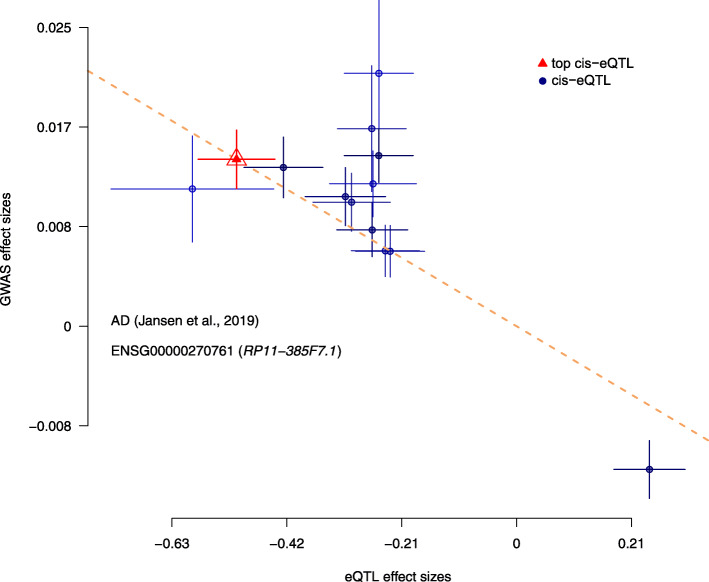
SMR Effect Plot for RP11-385F7.1 using Qi et al., 2018 cis-eQTL data and Jansen et al., 2019 meta-GWAS data. X-axis represents cis-eQTL effect sizes while the y-axis represents GWAS effect sizes

**Fig. 6:**
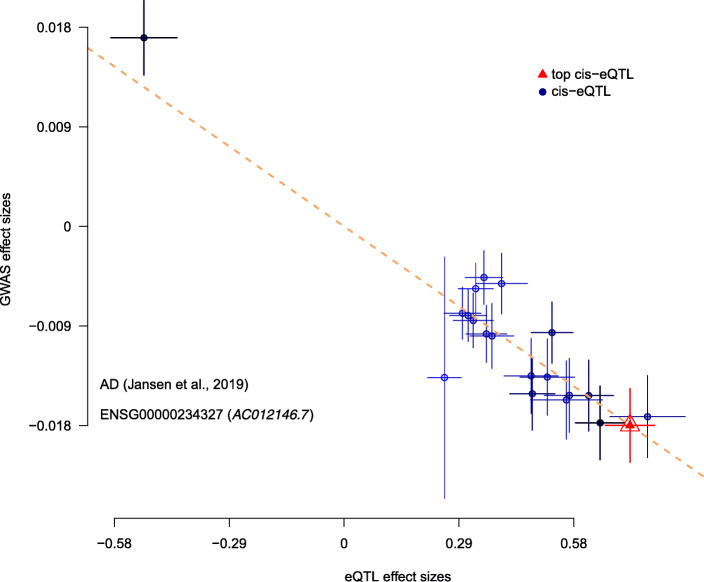
SMR Effect Plot for AC012146.7 using Qi et al., 2018 cis-eQTL data and Jansen et al., 2019 meta-GWAS data. X-axis represents cis-eQTL effect sizes while the y-axis represents GWAS effect sizes

**Fig. 7:**
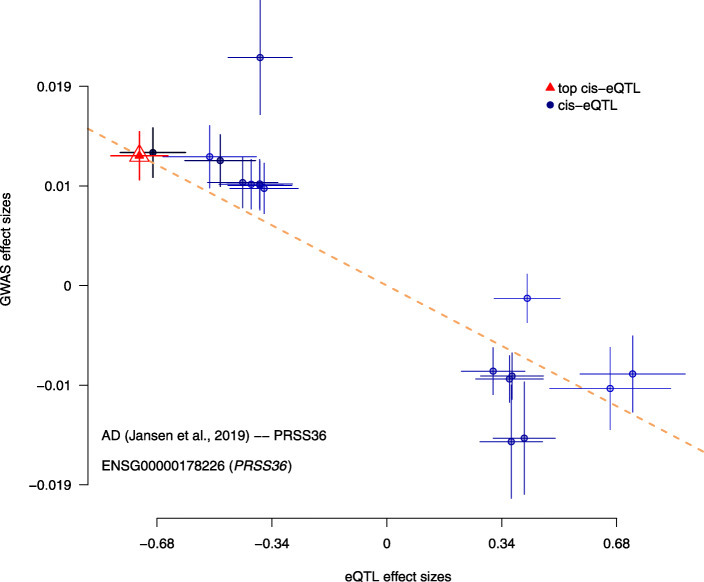
SMR Effect Plot for PRSS36 using Qi et al., 2018 cis-eQTL data and Jansen et al., 2019 meta-GWAS data. X-axis represents cis-eQTL effect sizes while the y-axis represents GWAS effect sizes

Furthermore, we also wished to confirm the directionality of the effects found via this SMR analysis between specific genes and our phenotype of AD. As such, we provide the effect plots in Figs. 5, 6, and 7. They show the correlation between the eQTL effect sizes and GWAS effect sizes for our novel findings (RP11-385F7.1, AC012146.7, and PRSS36) with the GWAS summary data sets from Jansen et al., 2019 and our single source of meta-analysis cis-eQTL data from Qi et al., 2018. Each plot shows the correlation between GWAS effect sizes and our set of meta-analysis cis-eQTL’s. In particular, we are comparing the effect sizes of SNPs (used for the SMR and the relevant HEIDI tests) from GWAS plotted against those for SNP’s from our meta-analysis cis-eQTL data. Notably, from these plots one can see the existence of negative correlations between our GWAS effect sizes and eQTL effect sizes in Figs. 5, 6, and 7.

## Discussion

In this section, we provide a brief discussion on our three novel findings to determine the larger context of their significance in AD. RP11-385F7.1 is a long intergenic non-coding RNA (LinkRNA) gene on Chromosome 6. According to the GTEx Portal’s page for this gene, although we have seen that this gene is decently expressed in the brain tissues, it is most strongly expressed in the kidneys and pituitary gland [15]. This locus has also been found by [16] to likely have a functional effect within AD, which corroborates the findings of this study.

PRSS36 is a protein-coding gene on chromosome 16. According to OMIM, it codes for Serine Protease 36, a protease that may be instrumental in hydrolyzing serine protease substrates. Additionally, a northern blot analysis shows a 5 kb transcript of this gene in fetal kidney and adult skeletal muscle, the liver, the placenta, and the heart [17]. To confirm if this gene’s native protein, serine protease 36, plays a role in AD, a search in the Open Targets Platform was performed. PRSS36 has been highlighted in [10] and [18] for its high genetic association with AD (*p*=4×10^−8^ in the former; *p*=3×10^−8^ in the latter.) This is the only one of our findings found in the Open Targets Platform; perhaps as these gene targets are studied more, more significant correlations may be found in the future.

AC012146.7 is another non-coding gene (specifically, processed transcript) located on chromosome 17. Not much is known about its function or clinical significance, though it is located near the protein coding genes USP6 and ZNF232 [19]. ZNF232 is a protein encoding gene that encodes for Zinc Finger Protein 232. Zinc finger proteins are involved in the regulation of several cellular processes, including transcriptional regulation, signal transduction, and DNA repair [20]. Meanwhile, USP6 encodes Ubiquitin-specific Peptidase 6, which is commonly associated with psuedosarcomatous fibromatosis and fasciitis [21].

With the above observations, these genes can be studied in more detail going forward. SMR-based replication studies can be performed in independent cohorts. The potential of these genes to serve as molecular targets for AD studies within specific tissues of the brain as determined by these causal analyses also warrants further biological investigations, potentially including but not limited to the analysis of brain-related functional data, brain ATAC, brain-related HiC, and brain-related pcHiC in an independent cohort. These additional analyses may demonstrate the regulatory mechanism by which these variants- and genes-of-interest act or elucidate an underlying function these variants play in AD pathogenesis.

Our approach using Summary-data-based Mendelian Randomization has allowed for the inclusion of independently collected and curated GWAS and cis-eQTL data. This has provided our study a significant amount of statistical power it may not have had otherwise due to the small number of samples that include AD diagnosis data, full genotyping data, and extensive gene expression data. Implementing an instrumental variables estimation using meta-anlaysis GWAS and eQTL data in particular has allowed us to analyze an unprecedented number of individuals in a very short amount of time. However, one limitation of our approach is that our implementation of the instrumental variable estimation has included the use of stringent Bonferroni method for multiple comparison correction. As a result, it is likely some significant signals were missed in our analyses. Alternatively, it may be possible to instead employ corrections based on the false-discovery rates (FDR) provided by the SMR analyses to determine significance in a less conservative fashion [22].

## Conclusions

We have performed an SMR analysis that integrated meta-analytic cis-eQTL summary statistics from GTEx, CMC, and ROS/MAP studies with three sets of meta-analysis GWAS results in AD. We aim to discover genes differentially expressed in AD for better understanding of the molecular mechanism of the disease. Our analysis identified twelve total gene probes (associated with twelve distinct genes) with a significant association with AD. Four of these genes survived a test of pleiotropy from linkage (the HEIDI test). One of the four genes, NDUFS2, has been previously reported as differentially expressed in the brain in the context of AD. The remaining three genes – RP11-385F7.1, PRSS36, and AC012146.7 – have not yet been reported differentially expressed in the brain in the context of AD. However, there exist prior studies suggesting some indirect connections between these genes and AD. Thus, further investigations, including performing SMR-based replication studies in independent cohorts and/or conducting molecular validation using brain-related tissues in AD research, may study these genes in more detail.

## Methods

### Genotyping reference data

To assist in checking the consistency of allele frequency and effect-allele information between the GWAS and eQTL datasets in each respective SMR analysis, the SMR program by default requires a reference panel of genetic data. In our analysis, we used the genome-wide genotyping data sourced from the Alzheimer’s Disease Neuroimaging Initiative (ADNI) database [23, 24]. This data is publicly accessible on the ADNI Data Archive at http://adni.loni.usc.edu/.

ADNI was launched in 2003 as a public-private partnership led by Principal Investigator Michael W. Weiner, MD to test whether serial MRI, PET, and biological markers can be combined with clinical and neuropsychological assessments to accurately measure the progression of mild cognitive impairment (MCI) and early AD. For more information about the ADNI project, please see [23, 24].

Participants were limited to individuals who were subjects of the ADNI cohort. To reduce the likelihood of population stratification effects, only non-Hispanic Caucasian participants were involved. As such, there were 1,576 individuals whose genotyping data were included. 521 of these individuals are healthy controls and the remaining 1,055 individuals are patients with AD or mild cognitive impairment (MCI, a prodromal stage of AD), and are all coded as cases in this study.

Genotyping data were quality-controlled, imputed using the 1000 Genomes Project reference genomes, and combined as described in [25, 26]. Briefly, genotyping was performed on all ADNI participants following the manufacturer’s protocol using blood genomic DNA samples and Illumina GWAS arrays (610-Quad, OmniExpress, or HumanOmni2.5-4v1) [27]. Quality control was performed in PLINK v1.90 [28] using the following criteria: 1) call rate per marker ≥95*%*, 2) minor allele frequency (MAF) ≥5*%*, 3) Hardy Weinberg Equilibrium (HWE) test P ≤1.0E-6, and 4) call rate per participant ≥95*%*. As a result, a total of 5,574,300 SNPs were included in our analysis.

### GWAS summary data

To ensure the highest levels of statistical power, we opted to utilize the results of large-scale meta-GWAS studies in AD in our analysis. As such, there are three best-known landmark AD GWAS analyses we examined in our study.

The first is a meta-analysis of 74,046 individuals which studied 7,055,881 directly genotyped or imputed SNPs, which summarized the results of the International Genomics of Alzheimer’s Project (IGAP) [9]. This project included 17,008 AD cases and 37,154 controls, which represent the synthesis of 4 previously published GWAS data sets and has found 11 loci newly associated with AD. Summary statistics from this study included SNP chromosome, position, and effect/non-effect allele information along with statistics summarizing GWAS linear regression results (i.e. effect size, standard error of this effect size, and the meta-analysis *p*-value using regression coefficients). The SMR analytical program also required frequency information for the effect alleles reported. As the IGAP chose to not share allele frequency data due to privacy concerns, however, we instead extracted this information using PLINK v1.90 [28] from the genotyping reference panel data discussed above. The summary statistics for the IGAP study can be found at https://www.niagads.org/datasets/ng00036. To maximize the power of our analyses, the most updated combined Stage 1 and Stage 2 data was used.

The second analysis used in this work conducts a meta-analysis that included clinically-diagnosed AD as well as AD-by-proxy, which included a total of 71,880 cases and 383,378 controls [10]. As [10] is not specifically an AD study, AD status of individuals in their cohort was determined by examining their family history. If one or more biological parents were diagnosed with late-onset AD sometime in their life, the individual (child) would be coded as AD-positive. This is possible given the strong genetic basis of AD. Given that this study did not/could not directly assess an individual’s AD status, AD results from this study have been termed ‘AD-by-proxy.’ AD-by-proxy has been shown to have very strong genetic ties to clinical AD with a *r*_*g*_=0.81; thus, individuals who have AD-by-proxy may be coded as ‘case’ individuals similar to those with a clinical AD diagnosis from a genetics standpoint. This greatly enlarges the number of individuals included in the study and thus increases statistical power. With this significantly larger data set, this analysis was able to identify 29 risk loci for AD. The summary statistics used for this study can be found at (https://ctg.cncr.nl/software/summary_statistics) under the heading ‘Summary statistics for Alzheimer’s dementia from Iris Jansen et al., 2019.’ Our analyses utilized the most updated version of the data, which was published in December 2019.

The third analysis used is also a meta-analysis [11]; this is a continuation of the first analysis noted above. In addition to expanding the population size from individuals of European descent to non-Hispanic Whites, this analysis uses a larger discovery sample which has implemented 17 new datasets, leading to a total *n*=21,982 with 41,944 cognitively normal controls. The main projects involved with this meta-analysis include the Alzheimer Disease Genetics Consortium (ADGC), Cohorts for Heart and Aging Research in Genomic Epidemiology Consortium (CHARGE), The European Alzheimer’s Disease Initiative (EADI), and Genetic and Environmental Risk in AD/Defining Genetic, Polygenic and Environmental Risk for Alzheimer’s Disease Consortium (GERAD/PERADES). The genotypic datasets were imputed using a 1,000 Genomes reference panel to include a total 36,648,992 SNP’s; 1,380,736 indels; and 13,805 structural variants; this analysis leads to the identification of five novel genome-wide loci associated with AD, two of which have also been found in the second analysis. The summary statistics can be found on NIAGADS at (https://www.niagads.org/datasets/ng00075). The most recent version of this data, which was published in February 2019, was used in the analysis.

### cis-eQTL summary data

cis-eQTL data used in this study was derived from a meta-analysis of cis-eQTL’s between independent brain and blood samples [29]. The exact meta-analysis cis-eQTL information in the format required by the SMR tool can be downloaded in full at https://bit.ly/3gRNbGC.

This study integrated eQTL information from multiple sources, including the GTEx project gene expression data derived from both the blood and ten separate brain tissues, CommonMind Consortium gene expression data derived from the dorsolateral prefrontal cortex, and ROS/MAP gene expression data. cis-eQTL’s, as defined by having the distance between a SNP and gene probe being less than 1 Mb, were chosen in favor of trans-eQTL data because trans-eQTL data was not available for most of the data sets chosen by the study.

In their study, due to the use of biomarkers from the blood as well as the brain from several different cohorts, Qi et al. quantitatively established the similarity of genetic effects at the top-associated cis-eQTLs between blood and brain-derived measures. They show the correlation of cis-eQTLs between brain and blood is fairly high, with *r*_*b*_≈0.79 between the GTEx WholeBlood and Hippocampus cis-eQTL’s, for instance. This allows for the integration of cis-eQTL’s taken from blood-derived tissues in our analysis.

Such a meta-analysis is extremely powerful due to the enlarged sample size of such an analysis. Previous analyses utilizing gene expression data from any one of these three sources alone, especially those that studied brain tissues, were somewhat hindered by the small sample sizes of each respective study. However, synthesizing these data sets and including the blood-based biomarkers from the GTEx project would allow for an adequately large and statistically powerful analysis. As such, it was important to determine if this could be properly done and if these individual cis-eQTL’s lead to similar conclusions despite being sourced from very different tissues. Fortunately, this was proven to be possible, as shown by a $\hat {r}_{b} = 0.70$ for cis-eQTL’s, which show that there is a high correlation between independent brain and blood samples, allowing for the combination of these cis-eQTL’s and our proposed analysis.

Given these reassurances, the meta-analysis of cis-eQTL data was performed with *n* ranging from 526 to 1194. The meta-analysis of these cis-eQTL’s has been calculated using a program called MeCS [8], which uses the summary-level cis-eQTL data provided from these three consortiums to perform meta-analyses of cis-eQTLs. In the MeCS calculation, cis-eQTL’s were selected based on a definition of locality limited to only SNP’s within 1 Mb of the gene probe in question, as defined above. More information can be found about MeCS, including a copy of the software, at https://cnsgenomics.com/software/smr/#MeCS.

### The SMR method

**Fig. 8:**
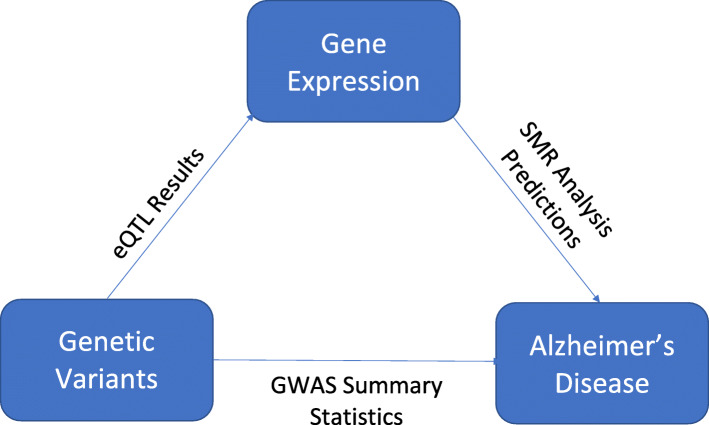
Flowchart outlining the instrumental variable procedure of SMR. Known relationships represented by eQTL between genetic variants and gene expression and GWAS between genetic variants and AD are represented by solid arrows. The gene expression - AD (causal) relationship that we are trying to establish via SMR is represented by a dotted-line arrow

Summary-data-based Mendelian Randomization (SMR) uses an instrumental variable estimation in order to accurately integrate independent GWAS and eQTL summary data. A diagram visualizing the vital relationships this approach utilizes is shown in Fig. 8. Briefly, an instrumental variable estimation can be used to better understand the correlation between an independent variable and a dependent variable, especially when our independent and dependent variables are endogenous [30]. Mendelian Randomization (MR) as a whole is a biological adaptation of this approach [31, 32].

The scientific basis of MR relies on a variant of the central dogma of biochemistry: the ideal that genetic variations (DNA) affect how certain genes are expressed (RNA), which in turn affect the proteins produced by the cell, potentially leading to changes on a systemic level (phenotype). It has been previously shown that if a specific genetic variant (i.e. one of the SNP’s studied in the meta-analysis cis-eQTL) were to affect the expression of a gene – a relationship potentially found via a cis-eQTL analysis [33] – then there will be differences in gene expression levels among individuals with different genetic ‘versions’ of the studied SNP (i.e. heterozygous versus homozygous dominant versus homozygous recessive). These differences, in turn, are analogous to the overexpression (in our case, positive AD diagnosis, assuming our SNP and gene are risk factors for AD) and/or suppression (a lack of a diagnosis) of the phenotype studied [8]. A MR analysis takes a very similar approach, in using a SNP as an instrumental variable to test the magnitude and presence of a causal effect of the expression of a specific gene on our outcome of interest. In principle, it is thus possible to use a MR approach to search for the genes at the loci of the SNP’s highlighted in our summary GWAS that have the highest functionality in AD. In finding highly significant/impactful gene probes, this analysis may lead to the discovery of certain genes that have yet to be declared differentially expressed in AD.

Up until recently, it was highly likely that in order to perform an accurate Mendelian Randomization approach, a full set of data involving GWAS, eQTL, and phenotype data for a large cohort was necessary to produce statistically robust results. With the work of Zhu et al. [8], it is now possible to perform a Mendelian Randomization using only summary data potentially using GWAS and eQTL data from different studies. Their approach makes this possible using a series of corrections and assumptions about the input data, which allows for maximum efficiency while implementing conservative screens that ensure only the most statistically significant correlations between gene expression and phenotype are highlighted.

First, as the given genetic variants are the primary bridge between the comparisons with phenotype and gene expression data, the program performs a quality-control effect allele frequency check to verify the SNP information used in both the eQTL and GWAS studies are congruous. Next, given the need for a significant SNP-eQTL relationship to exist in order to perform the Mendelian Randomization analysis as mentioned above, only cis-eQTL’s (as defined by the standard 1 Mb radius from the gene probe) with a top *P*_*eQTL*_≤5×10^−8^ are included for the SMR analysis. Furthermore, SNP’s with eQTL minor, effect, and/or GWAS allele frequencies <0.01 were also removed. Then, only SNP’s with eQTL *p*-values that survive a Bonferroni-corrected p threshold as defined by the number of SMR calculations ran per command are fully analyzed. Lastly, to correct for linkage disequilibrium scattering results, SNP’s with a *r*^2^>0.90 or *r*^2^<0.05 with the top SNP for that cis-eQTL are excluded, with one result of every pair of SNP’s that satisfy these LD requirements also being excluded.

With this procedure, it is possible to gain insight as to the significance of certain genes relevant to AD. However, an SMR analysis is not all that is needed to confirm the causal relationship between gene expression and phenotype.

Of note, a strong association in a SMR test doesn’t necessarily mean that gene expression and the trait in question are both directly affected by the same underlying genetic variant. It is possible that the association is due to the top associated cis-eQTL variant being in linkage disequilibrium with two separate variants, one of which may influence gene expression and the other which may affect our phenotypic outcome. This type of linkage is significantly less powerful than the pleiotropic relationships we wish to find instead.

To differentiate between the pleotropic relationships we wish to find and the linkage relationships we wish to avoid, Zhu et al. [8] created the Heterogeneity in Dependent Instruments (HEIDI) test. This technique specifically tests against the null hypothesis that there is a single null variant, which is biologically equivalent to testing if there is heterogeneity in the effect sizes estimated for SNP’s in the cis-eQTL region of interest. Since the HEIDI test has been shown to help identify variants that are most likely to have a strong effect on both gene expression and our AD phenotype, it was used to distinguish pleiotropy from linkage in the context of our analyses, similar to the work presented in [8]. Of course, variants highlighted by the SMR technique and HEIDI test also warrant further biological investigation.

We have performed an SMR analysis that integrated meta-analytic cis-eQTL summary statistics from GTEx, CMC, and ROS/MAP studies with three sets of meta-analysis GWAS results in AD. We aim to discover genes differentially expressed in AD for better understanding of the molecular mechanism of the disease. Our analysis identified twelve total gene probes (associated with twelve distinct genes) with a significant association with AD. Four of these genes survived a test of pleiotropy from linkage (the HEIDI test). One of the four genes, NDUFS2, has been previously reported as differentially expressed in the brain in the context of AD. The remaining three genes – RP11-385F7.1, PRSS36, and AC012146.7 – have not yet been reported differentially expressed in the brain in the context of AD. However, there exist prior studies suggesting some indirect connections between these genes and AD. Thus, further investigations, including performing SMR-based replication studies in independent cohorts and/or conducting molecular validation using brain-related tissues in AD research, may study these genes in more detail.

## Data Availability

Summary GWAS information is publicly available from [9–11]. Meta-eQTL information is publicly available from [29].

## Abbreviations

GWAS: Genome-Wide Association Study
AD: Alzheimer’s Disease
SMR: Summary-data-based Mendelian Randomization
(cis-/trans-/meta-)eQTL: (cis-/trans-/meta-) Expression Quantitative Trait Locus
GTEx: Genotype-Tissue Expression
CMC: CommonMind Consortium
ROS/MAP: Religious Orders Study and Rush Memory and Aging Project
ADNI: Alzheimer’s Disease Neuroimaging Initiative
HEIDI: Heterogeneity in Dependent Instruments
HWE: Hardy Weinberg Equilibrium
SNP: Single Nucleotide Polymorphism
MAF: Minor Allele Frequency
IGAP: Internaional Genomics of Alzheimer’s Project
ADGC: Alzheimer Disease Genetics Consortium
CHARGE: The Cohorts for Heart and Aging Research in Genomic Epidemiology
EADI: The European Alzheimer’s Disease Initiative
GERAD/PERADES: The Genetic and Environmental Risk in Alzheimer’s Disease/Defining Genetic, Polygenic and Environmental Risk for Alzheimer’s Disease
NIAGADS: The National Institute on Aging Genetics of Alzheimer’s Disease Data Storage Site
Mb: megabase(s)
MeCS: Meta-analysis of cis-eQTL in Correlated Samples
MR: Mendelian Randomization
LD: Linkage Disequilibrium
